# Supplementary material for: Improving the Pharmacological Properties of Ciclopirox for Its Use in Congenital Erythropoietic Porphyria
Source: J Pers Med. 2021 May 28;11(6):485. doi: 10.3390/jpm11060485 (PMC8230281; doi:10.3390/jpm11060485)
Supplement: Supplementary file 1 [file jpm-11-00485-s001.zip › jpm-1197461-supplementary.pdf]

SUPPLEMENTARY MATERIAL

Improving the pharmacological properties of ciclopirox for its use in congenital erythropoietic porphyria

Ganeko Bernardo-Seisdedos[1], Jorge M. Charco[1], Itxaso SanJuan[2], Sandra García-Martínez[1], Pedro Urquiza[2], Hasier Eraña[1], José M Mato[2], Joaquín Castilla[1,2] and Oscar Millet[1,2]

[1]ATLAS Molecular Pharma S. L. Parque Tecnológico de Vizcaya, Ed. 800, Derio 48160, Spain.

[2]CIC bioGUNE, BRTA, Parque Tecnológico de Vizcaya, Ed. 800, Derio 48160, Spain.

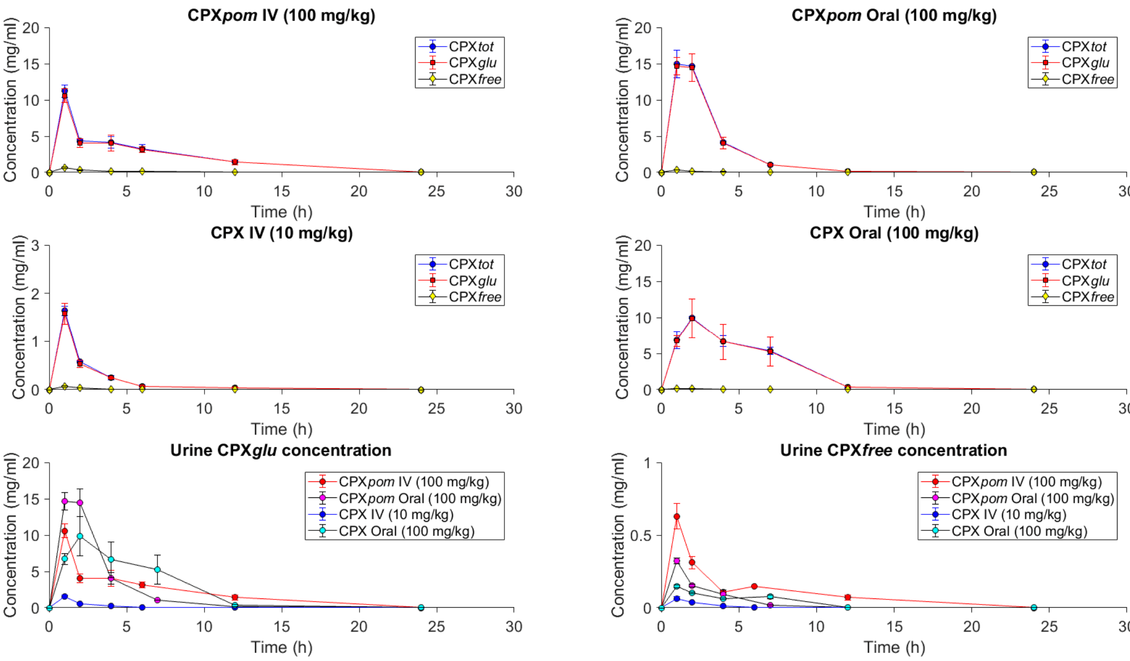

Figure S1. Urine CPX concentrations (µg/mL) as a function of time, as determined by NMR spectroscopy. Mean serum CPX concentrations are shown as square whereas the S.D. values are expressed in bars.

**CPX*pom***								**CPX**							
IV [100 mg/kg]				Oral [100 mg/kg]				IV [10 mg/kg]				Oral [100 mg/kg]			
h	CPX_{tot}	CPX_{glu}	CPX_{free}	h	CPX_{tot}	CPX_{glu}	CPX_{free}	h	CPX_{tot}	CPX_{glu}	CPX_{free}	h	CPX_{tot}	CPX_{glu}	CPX_{free}
0	0.00 ± 0.0	0.00 ± 0.0	0.00 ± 0.0	0	0.00 ± 0.0	0.00 ± 0.0	0.00 ± 0.0	0	0.00 ± 0.0	0.00 ± 0.0	0.00 ± 0.0	0	0.00 ± 0.0	0.00 ± 0.0	0.00 ± 0.0
1	42.8 ± 0.5	33.7 ± 3.3	9.06 ± 0.0	1	45.3 ± 3.7	41.3 ± 7.66	3.91 ± 0.6	1	5.80 ± 0.3	4.98 ± 0.0	0.82 ± 0.2	1	43.1 ± 1.3	39.4 ±13.7	3.66 ± 0.6
2	33.2 ± 3.8	26.0 ± 5.2	7.16 ± 0.6	2	36.2 ± 4.0	33.4 ± 0.7	2.80 ± 0.2	2	3.73 ± 0.1	2.52 ± 0.4	1.21 ± 0.0	2	30.9 ± 12.0	27.9 ± 0.1	3.01 ± 0.2
4	23.2 ± 0.9	17.8 ± 3.8	5.38 ± 1.2	4	12.5 ± 0.4	10.1 ± 3.2	2.38 ± 0.2	4	1.84 ± 0.6	1.51 ± 0.1	0.33 ± 0.0	4	20.5 ± 6.2	17.8 ± 0.8	2.68 ± 0.1
6	12.2 ± 0.1	11.2 ± 1.7	0.92 ± 0.0	7	5.12 ± 0.4	4.09 ± 0.5	1.03 ± 0.4	6	0.66 ± 0.2	0.66 ± 0.1	0.00 ± 0.0	7	11.2 ± 1.2	10.2 ± 2.1	1.06 ± 0.4
12	2.06 ± 0.7	0.90 ± 0.4	1.16 ± 0.2	12	2.65 ± 0.0	1.97 ± 0.2	0.68 ± 0.2	12	0.10 ± 0.0	0.10 ± 0.0	0.00 ± 0.0	12	6.37 ± 2.6	5.18 ± 1.0	1.20 ± 0.1
24	0.00 ± 0.0	0.00 ± 0.0	0.00 ± 0.0	24	0.93 ± 0.0	0.18 ± 0.0	0.00 ± 0.0	24	0.00 ± 0.0	0.00 ± 0.0	0.00 ± 0.0	24	0.50 ± 0.1	0.50 ± 0.1	0.00 ± 0.0
48	0.00 ± 0.0	0.00 ± 0.0	0.00 ± 0.0	48	0.00 ± 0.0	0.00 ± 0.0	0.00 ± 0.0	48	0.00 ± 0.0	0.00 ± 0.0	0.00 ± 0.0	48	0.00 ± 0.0	0.00 ± 0.0	0.00 ± 0.0

Table S1. Serum Ciclopirox concentrations (µg/ml)

**CPX*pom***								**CPX**							
IV [100 mg/kg]				Oral [100 mg/kg]				IV [10 mg/kg]				Oral [100 mg/kg]			
h	CPX_{tot}	CPX_{glu}	CPX_{free}	h	CPX_{tot}	CPX_{glu}	CPX_{free}	h	CPX_{tot}	CPX_{glu}	CPX_{free}	h	CPX_{tot}	CPX_{glu}	CPX_{free}
0	0.00 ± 0.0	0.00 ± 0.0	0.00 ± 0.0	0	0.00 ± 0.0	0.00 ± 0.0	0.00 ± 0.0	0	0.00 ± 0.0	0.00 ± 0.0	0.00 ± 0.0	0	0.00 ± 0.0	0.00 ± 0.0	0.00 ± 0.0
1	11.21 ± 0.8	10.58 ± 0.9	0.63 ± 0.1	1	14.95 ± 1.9	14.63 ± 1.2	0.32 ± 0.0	1	1.64 ± 0.1	1.58 ± 0.2	0.06 ± 0.0	1	6.86 ± 1.2	6.71 ± 0.8	0.15 ± 0.0
2	4.35 ± 0.2	4.04 ± 0.6	0.31 ± 0.0	2	14.63 ± 0.1	14.48 ± 1.9	0.15 ± 0.0	2	0.57 ± 0.0	0.53 ± 0.0	0.04 ± 0.0	2	9.97 ± 0.2	9.87 ± 2.7	0.10 ± 0.0
4	4.17 ± 0.8	4.06 ± 1.1	0.11 ± 0.0	4	4.14 ± 0.2	4.05 ± 0.8	0.09 ± 0.0	4	0.25 ± 0.0	0.24 ± 0.0	0.01 ± 0.0	4	6.67 ± 0.8	6.60 ± 2.4	0.06 ± 0.0
6	3.29 ± 0.5	3.14 ± 0.4	0.15 ± 0.0	7	1.09 ± 0.2	1.07 ± 0.0	0.02 ± 0.0	6	0.06 ± 0.0	0.06 ± 0.0	0.00 ± 0.0	7	5.36 ± 0.5	5.28 ± 2.0	0.08 ± 0.0
12	1.49 ± 0.2	1.42 ± 0.4	0.07 ± 0.0	12	0.18 ± 0.0	0.18 ± 0.0	0.00 ± 0.0	12	0.04 ± 0.0	0.04 ± 0.0	0.00 ± 0.0	12	0.31 ± 0.0	0.31 ± 0.1	0.00 ± 0.0
24	0.06 ± 0.0	0.06 ± 0.0	0.00 ± 0.0	24	0.02 ± 0.0	0.02 ± 0.0	0.00 ± 0.0	24	0.00 ± 0.0	0.00 ± 0.0	0.00 ± 0.0	24	0.08 ± 0.0	0.07 ± 0.0	0.00 ± 0.0
48	0.00 ± 0.0	0.00 ± 0.0	0.00 ± 0.0	48	0.00 ± 0.0	0.00 ± 0.0	0.00 ± 0.0	48	0.00 ± 0.0	0.00 ± 0.0	0.00 ± 0.0	48	0.03 ± 0.0	0.03 ± 0.0	0.00 ± 0.0

Table S2. Urine Ciclopirox concentrations (mg/ml).

REF		Non-compartmental Analysis			
**DRUG**		**CPX*pom***		**CPX**	
**VIA**		IV	Oral	IV	Oral
**DOSE**	mg/kg	100.00	100.00	10.00	100.00
**Weight**	kg	0.02	0.02	0.02	0.02
**DM**	mg	2.00	2.00	0.20	2.00
**Cmax**	mg/L	51.21	52.87	8.56	43.40
**Tmax**	h	0	1.3	0	0.86
**T_half**	h	3.34	2.45	1.75	3.26
**AUC 0 → 12h**	mg*(h/L)	210.99	149.80	2.40	199.20
**AUMC 0 → 12h**	mg*(h/L)	704.35	502.54	45.01	812.64
**AUC_half 0 → 12h**	h	2.64	2.28	1.61	3.13
**MRT 0—> 12h**	h (AUMC/AUC)	3.34	2.35	2.21	4.08
**k_el (λ_z)**	h-1 (0.693/t1/2)	0.21	0.28	0.40	0.21
**K_el**	h-1 (1/MRT)	0.30	0.30	0.45	0.25
**Cl**	L/h	0.01	0.01	0.01	0.01
**Vss**	L	0.03	0.04	0.02	0.04
**F (Oral vs IV) 0 → 12h**	%	71		98	
**CPX_{FREE}**	**(%)**	< 22.0	< 11.8	< 21.4	< 10.4
**AUC_{URINE} 0 → 24h**	g*(h/L)	140.06	139.02	11.80	126.86

Table S3. Serum CPX pharmacokinetic parameters in mice following single IV or oral doses of CPX*pom* and CPX.
